# Individual Pharmacotherapy Management (IPM)—IV: Optimized Usage of Approved Antimicrobials Addressing Under-Recognized Adverse Drug Reactions and Drug-Drug Interactions in Polypharmacy

**DOI:** 10.3390/antibiotics11101381

**Published:** 2022-10-09

**Authors:** Ursula Wolf, Henning Baust, Rüdiger Neef, Thomas Steinke

**Affiliations:** 1 Pharmacotherapy Management, University Hospital Halle (Saale), Martin Luther University Halle-Wittenberg, 06120 Halle (Saale), Germany; 2 University Clinic for Anesthesiology and Operative Intensive Care Medicine, University Hospital Halle (Saale), Martin Luther University Halle-Wittenberg, 06120 Halle (Saale), Germany; 3 Department of Orthopedics, Trauma and Reconstructive Surgery, Division of Geriatric Traumatology, University Hospital Halle (Saale), Martin Luther University Halle-Wittenberg, 06120 Halle (Saale), Germany; 4 Clinic for Anesthesiology, Intensive Care Medicine and Pain Therapy, Carl-von-Basedow-Klinikum Saalekreis, 06127 Merseburg, Germany

**Keywords:** antimicrobial, antibiotics, antifungals, adverse drug reaction (ADR), drug-drug interaction (DDI), polypharmacy, multimorbidity, intensive care patients, traumatology, elderly patients, organ failure, multi-organ failure, drug safety, patient safety

## Abstract

Antimicrobial therapy is often a life-saving medical intervention for inpatients and outpatients. Almost all medical disciplines are involved in this therapeutic procedure. Knowledge of adverse drug reactions (ADRs) and drug-drug interactions (DDIs) is important to avoid drug-related harm. Within the broad spectrum of antibiotic and antifungal therapy, most typical ADRs are known to physicians. The aim of this study was to evaluate relevant pharmacological aspects with which we are not so familiar and to provide further practical guidance. Individual pharmacotherapy management (IPM) as a synopsis of internal medicine and clinical pharmacology based on the entirety of the digital patient information with reference to drug information, guidelines, and literature research has been continuously performed for over 8 years in interdisciplinary intensive care and trauma and transplant patients. Findings from over 52,000 detailed medication analyses highlight critical ADRs and DDIs, especially in these vulnerable patients with polypharmacy. We present the most relevant ADRs and DDIs in antibiotic and antifungal pharmacology, which are less frequently considered in relation to neurologic, hemostaseologic, hematologic, endocrinologic, and cardiac complexities. Constant awareness and preventive strategies help avoid life-threatening manifestations of these inherent risks and ensure patient and drug safety in antimicrobial therapy.

## 1. Introduction

Antimicrobial therapy often means a life-saving medical intervention for in-hospital and outpatients, and almost all medical disciplines are involved in this therapeutic regimen. Knowledge of adverse drug reactions (ADRs) and drug-drug interactions (DDIs) is important to avoid drug-related harm, as severe organ damage or life-threatening conditions can already occur with dosing due to drug-drug-inhibited metabolism. With respect to the expanding elderly patient population with polypharmacy, the almost rapidly increasing drug availabilities, and administration by different specialists in multimorbidity, ARDs and DDIs are turning into a major health problem worldwide. An ADR is “a response to a drug which is noxious and unintended and which occurs at doses normally used in man for prophylaxis, diagnosis, or therapy of disease or for the modification of physiologic function” (WHO 1972), which has already been defined by the WHO for 50 years [1]. A further, more clarified terminology refers to an ADR as “an appreciably harmful or unpleasant reaction resulting from an intervention related to the use of a medicinal product; adverse effects usually predict hazard from future administration and warrant prevention, or specific treatment, or alteration of the dosage regimen, or withdrawal of the product”, and classical type A (augmented) and type B (bizarre) ADRs display variable patterns depending on age, sex, race, diseases, drug category, route of administration, and DDIs, as well as the individual genotypic profile, which is a further determinant for the manifestation of ADRs [2]. The classification into six ADR types (A, B, C, D, E, F) aims to differentiate dose-related (augmented), non-dose-related (bizarre), dose-related and time-related (chronic), time-related (delayed), withdrawal (end of use), and failure of therapy (failure) ADRs. According to the directive 2001/83/EC of the European Parliament and the 2001 Council on the Community code relating to medicinal products for human use, reported in the Guideline on good pharmacovigilance practices (GVP)-Annex I (Rev 4), an ADR is currently defined as: “A response to a medicinal product which is noxious and unintended. Response in this context means that a causal relationship between a medicinal product and an adverse event is at least a reasonable possibility. An adverse reaction, in contrast to an adverse event, is characterized by the fact that a causal relationship between a medicinal product and an occurrence is suspected. For regulatory reporting purposes, if an event is spontaneously reported, even if the relationship is unknown or unstated by the healthcare professional or consumer as primary source, it meets the definition of an adverse reaction. Therefore all spontaneous reports notified by healthcare professionals or consumers are considered suspected adverse reactions, since they convey the suspicions of the primary sources, unless the primary source specifically state that they believe the event to be unrelated or that a causal relationship can be excluded. Adverse reactions may arise from use of the product within or outside the terms of the marketing authorization or from occupational exposure. Use outside the marketing authorization includes off-label use, overdose, misuse, abuse and medication errors [3].

As there are different definitions and categorizations for ADRs, which subsume DDIs [4,5,6], this may further complicate the predicament in the daily routine. Rather, the ADRs being increasingly reported post-marketing with varying drug co-medications may actually represent the result of so-far unknown pharmacokinetic or pharmacodynamic DDIs. In terms of our approach to management, the distinction between an inherently drug-associated ADR and a DDI resulting from combination therapy should always be clear for guiding physicians to avoid critical combinations, in particular. Any drug can cause ADRs to potentially manifest with a variety of clinical effects. Within the broad spectrum of antibiotic and antifungal therapy, most typical ADRs are or should be known to physicians. ADRs account for a high degree of hospitalization, up to 12%, and for a high rate of morbidity and mortality, ranking among the top 10 leading causes of death and illness in developed countries with enormous socioeconomic costs [7,8,9,10,11], irrespective of the presumably high number of unreported cases. This applies, especially, to antibiotics and intensive care patients [12,13]. In order not to confuse ADRs and DDIs with new disease symptoms and, thus, initiate an escalating spiral of therapeutic counter-regulation, the knowledge of specific ADRs is crucial, especially in intensive care medicine and in the polypharmacy of the elderly, who are particularly vulnerable in this context because of their frequent pre-existing severe or multimorbid conditions. Way back in 1997, Rochon and Gurwitz described the risks associated with the prescribing cascade and, in this context, called for the need to optimize drug treatment in the elderly patients most affected; in addition, a further distinction has been made bet- ween prescribing cascades that are sometimes appropriate, but must be regularly re-evaluated and documented, and problematic prescribing cascades [14,15]. Misinterpretation of an ADR as a new symptom or exacerbation of the underlying disease is the beginning of the prescribing cascade, prevalent in patients who already have extensive polypharmacy and impaired organ function and are, therefore, particularly susceptible to ADRs and DDIs. Today, 25 years later, the problem has become aggravated as the drugs available to treat an almost typical elderly multimorbid patient, e.g., with manifest hypertension, diabetes, heart failure, and COPD or urologic conditions, have become more numerous and diverse, physicians increasingly specialize, and the different disciplines do not even seem to be aware of the ADRs or DDIs of drugs from the other medical specialties. On top of this, changing organ functions of an intensively ill patient and, in addition, the complementary mechanical organ replacement procedures either temporarily or chronically influence the dosing regime and require very close monitoring of laboratory parameters in patients, often in life-threatening conditions.

Since it can be difficult to differentiate ADRs and DDIs from disease progression, for example, in the presence of pre-existing single- or multi-organ hypoxic failure, it is particularly important to focus on ADRs and DDIs from the onset of drug use, although in rare cases these may still occur even after drug discontinuation. The aim of this study was to highlight relevant pharmacological ADR and DDI aspects of antimicrobials administered in the hospital setting, which are not so widely known, and to provide further practical guidance from lessons learned based on long-term interdisciplinary experience with individual pharmacotherapy management (IPM).

## 2. Results

**Figure 1:**
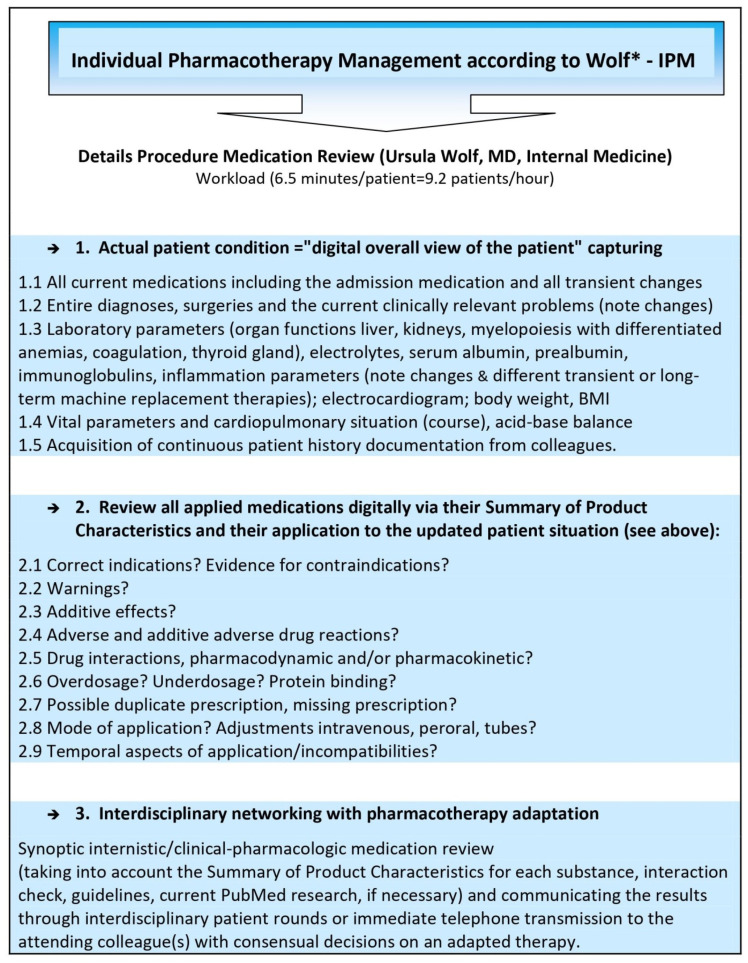
Comprehensive, reproducible IPM based on the hospital’s digital patient record. * Comprehensive, digitally based IPM, developed, implemented, and practiced by Ursula Wolf, MD, Head of the Pharmacotherapy Management Department, specialist in internal medicine, and expertised in clinical pharmacology, who performed >52,000 individual medication reviews.

Findings from over 52,000 detailed medication analyses continuously performed for over 8 years in interdisciplinary intensive care, transplant, and trauma patients highlight critical ADR and DDI risks, especially in these vulnerable patients with polypharmacy and/or with concomitant organ deterioration. This is where our individual pharmacotherapy management (IPM) (Figure 1) has proven very reliable and valuable in identifying and targeting drug-associated risks at the earliest stage. The ability to have the most comprehensive digital view of the patient as the basis for IPM is the essential prerequisite for adjusting each drug precisely for the individual patient’s medical condition as captured via metabolism and/or excretion depending on the specific mode of degradation of each drug, and always with the additional targeted focus on associated DDIs. Reading the Summary of Product Characteristics (SmPC) for each drug of an extensive drug list and, additionally, checking all pharmacokinetic and pharmacodynamic interactions between the drugs means an almost unmanageable procedure in the context of the time pressure of today’s physicians’ working conditions. We present the essence of often under-recognized but critical aspects of today’s typical drug combinations related to antimicrobials to link the almost unmanageable set of risk aspects of ADRs and DDIs in extensive polypharmacy to everyday clinical realities and to enable the sharing of eight years of daily IPM experience for the most critical issues. We explicitly outline the relevant risks of ADRs and DDIs in the clinical pharmacology of antimicrobial agents that are less frequently addressed adequately in terms of neurologic, hemostaseologic, hematologic, endocrinologic, and cardiac complexities.

The IPM strategy, which aims to assess each drug as comprehensively as possible and which accounts for the entire patient’s very acute clinical situation simultaneously, is shown in Figure 1. The first data on the preventative impact of this significantly effective IPM with a 90.2% reduction in delirium has been published previously [16]. Focusing on the entire patient condition with all of the information from the fully digitally available records proved to be a crucial and fundamental requirement for performing this extensive, individualized medication analysis, considering the respective, often abruptly changing, acute status in the ICU patients. The IPM process uncovers relevant complexities in almost all different groups of medications, and especially in polypharmacy, that have apparently been insufficiently addressed or not considered at all. Not infrequently, these manifested ADRs or DDIs were the reason for further deterioration of the clinical condition of the patient and his transfer to the ICU before application of IPM. Even therapeutic drug monitoring in antibiotics cannot guarantee drug safety, as it does not eliminate ARDs that are associated with regular dosing already or DDIs related to interacting co-medication. Despite knowledge of the often rapid alterations in renal function or metabolic rate due to DDIs or hepatic dysfunction, especially in multimorbid elderly patients and in transplant and intensive care patients with polypharmacy, immediate concomitant dose adjustment of the antibiotic regimen to fine-tune the daily dose is required more often than it is practiced. For this purpose, and to differentiate from ADRs and DDIs, close laboratory monitoring of these organ functions is mandatory.

Thus, knowledge gaps about ADRs and DDIs within the outpatient medication lists or in combination with in-hospital drugs have been identified repeatedly from more than 52,000 medication reviews performed regularly as part of IPM and therapeutic drug monitoring (TDM). Their presentation in this selection serves as an informative practice support for therapeutic application. Hospitalized patients with reviewed medication included elderly patients >70 years of age from the traumatology department, all patients in the operative ICUs, in the organ and stem cell transplantation departments, and those in further disciplines within the UKH; the patients‘ entire ambulatory drug therapy was mostly covered. This additional IPM effort intends to identify measures to improve clinicians’ recognition of these DDIs and prevent unwarranted ADRs of antimicrobial agents.

Being familiar with ADRs is essential to prevent drug-induced harm. Within the broad spectrum of antibiotic and antifungal therapy, physicians are aware of most typical ADRs. Thus, explicitly for the assessed relevant pharmacological issues in antimicrobial therapy that are less commonly considered, we provide further practical devices.

Our findings, in terms of relevant complexities in antimicrobial pharmacology that are less frequently considered, are presented in Table 1. They refer to ADRs and DDIs often recognized within a polypharmacy regimen or in patients with pre-existing organ deterioration.

### 2.1. Anidulafungin

Patients who experience elevated liver enzyme levels during anidulafungin therapy must be monitored for worsening liver function, and continuation of anidulafungin therapy must be subjected to a rigorous benefit–risk assessment. Hypokalemia and hyperglycemia, as well as headache and risk of convulsions, have to be considered [17].

### 2.2. Carbapenems

The rapid onset of extensive decline in blood levels of valproic acid when co-administered with carbapenem agents results in a 60–100% decrease in valproic acid levels in about two days [15]. This combination therapy is not manageable and must be avoided, and there are enough antiepileptic alternatives.

Neurologic/psychologic symptoms including hallucinations, drowsiness, dizziness, somnolence, and headache are ADRs to be aware of, and more rare symptoms comprise myoclonus, confusional states, seizures, paresthesias, encephalopathia, focal tremor, impaired sense of taste, and hypotension [18]. Seizures have been reported during treatment with carbapenem, including meropenem [18]. Our impression is that older patients are especially more at risk, often because of other concomitant diseases, cerebrovascular concerns, and medications that additionally increase the risk of seizures. Serum magnesium should be in the upper normal range and, if the risk is correspondingly high, such as after a stroke, concomitant medication with a similar seizure potential should be avoided as far as possible. With carbapenem, attention needs to be paid to thrombocytosis and thrombocytopenia. We observed high-grade thrombocytosis with carbapenem in the therapeutic course that started with onset, and if there was no contraindication, but risk related to thrombocytosis as in coronary patients, we temporarily applied acetylsalicylic acid (ASA) in a low dose. A differential diagnosis of thrombocytosis in antibiotic therapy remains, of course, a reactive thrombopoiesis in the recovery phase of an infection or a concomitant use of cortisone or special affections of the spleen.

### 2.3. Cotrimoxazole and Rifampicin

Not infrequently, hyperkalemia and hyponatremia are induced, necessitating simultaneous serum electrolyte monitoring.

Rifampicin decreases the effect of all currently available direct oral anticoagulants (DOACs) by inducing CYP3A4 and p-glycoprotein (P-gp); therefore, concomitant use is contraindicated. The same applies to rifampicin with calcineurin inhibitors and mammalian target of rapamycin inhibitors (mTORIs), where a dose elevation of the immunosuppressants must be assured under close TDM to prevent transplant rejection.

Typically, an inducer effect lasts a few days after discontinuation of the drug, and also manifests itself only maximally after a few days from the start of therapy in contrast to inhibitory metabolic disorders. This always has to be considered, for example, in the timing of TDM of immunosuppressants for contemporary dose adaptation [19].

### 2.4. Daptomycin

Daptomycin can cause anxiety, insomnia, dizziness, and headache. Use may result in false prolongation of prothrombin time (PT) and elevation of the international normalized ratio (INR) with certain recombinant thromboplastin reagents. Other drugs associated with myopathy, such as statins, should be temporarily discontinued; creatine phosphokinase (CPK) levels must be measured at baseline and at regular intervals. Eosinophilic pneumonia is a potential side effect of daptomycin. Daptomycin is one of the still-rare group of drugs whose bioavailability has been studied in relation to obesity. Obesity increases the area under the curve (AUC0-∞) of daptomycin [20].

### 2.5. Fluoroquinolones

Simultaneous administration with tube feeds is not recommended due to the minerals, and explicitly, divalent cations. There are interactions with antacids, iron, zinc, magnesium, sucralfate, calcium, didanosine, oral nutritional solutions, and dairy products. Ciprofloxacin should be administered 2 h before or at least 4 h after these products [21].

Systemic and inhaled fluoroquinolones may contribute to an increased risk of aneurysm, aortic dissection, and tendon rupture, but also of valvular regurgitation/insufficiency. A careful risk–benefit assessment is required, and other therapeutic options should be considered in patients at increased risk due to predisposing factors such as existing valvular heart disease, endocarditis, connective tissue diseases, hypertension, rheumatoid arthritis, and infectious arthritis [22].

### 2.6. Linezolid

Clinical use of linezolid in co-medication with serotonergic agents such as the antidepressant group of selective serotonin reuptake inhibitors (SSRIs) and serotonergic opioids is contraindicated because of the risk of serotonin syndrome. Linezolid is an antibiotic agent with nonspecific MAO-inhibitory activity and a predisposing risk for malignant neuroleptic syndrome. Therefore, it is important to know that excessive amounts of foods and beverages high in tyramine should be avoided, given the potential for significant pressure reactions [Lit]. A risk of myelosuppression (including anemia, leucopenia, pancytopenia, and thrombocytopenia) and lactate acidosis as ADRs needs to be considered with linezolid [23].

### 2.7. Similarities of Macrolides and Azoles concerning Severe DDIs

The combination of most macrolides or azoles with the widespread use of statins must be viewed very critically [24]. HMG-CoA reductase inhibitor (statin) ADRs such as myositis and rhabdomyolysis are often dose dependent. The bioavailability of the statins atorvastatin, lovastatin, and simvastatin, when co-administered with CYP3A44 inhibiting antimicrobials, increases, as do their ADRs. In this context, pravastatin, rosuvastatin, and fluvastatin are more inert due to their different metabolic and elimination pathways.

With macrolides or azoles, such as erythromycin, ketoconazole, or voriconazole, rhabdomyolysis and severe hepatic dysfunction have been observed to occur suddenly, especially with propofol plus amiodarone, which is also due to the marked, opposing CY3A4 inhibition of metabolism. This is essential to know because amiodarone itself can induce tachyarrhythmias, which, at the same time, evolve from the QT prolongating potential risk of most macrolides and azoles. These combinations aggravate and prolong the dangerous situation of the metabolism and toxicity of any drug involved via CYP3A4 metabolism and/or P-gp transport. Increasing hepatic dysfunction and even organ failure can rapidly escalate under these circumstances as the drugs become increasingly unmetabolized and cumulatively manifest their own hepatotoxic risks. These ADRs can accumulate into multi-organ failure, particularly in the presence of pre-existing renal failure. However, hepatic failure can be reversible with timely targeted drug discontinuation. Withdrawal of amiodarone often results in the timely resolution of the sometimes life-threatening problem. With regard to rhabdomyolysis, to prevent renal failure, we would like to point out that besides drug withdrawal or dose adjustment, the earliest administration of bicarbonate is recommended, always with reference to the simultaneously monitoring of adequate respiratory capacity, the partial pressure of carbon dioxide (pCO2), and the acid–base balance.

Fentanyl and buprenorphine must be withdrawn with the onset of macrolides or azoles because of the risk of, among other consequences, respiratory depressant effects due to decreased metabolism [25].

All currently approved DOACs are contraindicated with most macrolides (notice the metabolic properties of three different groups) and azoles because they increase the risk of hemorrhages due to the decreased DOAC degradation by inhibited P-gp and/or CYP3A4 [26]. The same applies to ticagrelor metabolism [27], which is also a substrate of P-gp and CYP3A4, a fact that is often not taken into account postinterventionally in cardiology patients and is further aggravated by concomitant hepatic dysfunction with reduced metabolic capacity, e.g., related to right-sided heart failure.

Both macrolides and azoles interact with several antiviral agents; in this context, and with respect to its current frequent use in the contemporary COVID-19 pandemic, we refer to remdesivir. Although remdesivir is a substrate of the metabolizing enzymes CYP2C8, CYP2D6, and CYP3A4, as well as a substrate of the transporters for organic anion-transporting polypeptide 1B1 (OATP1B1) and P-gp, no clinical interaction studies have been performed with remdesivir [28]. Concomitant administration of potent inhibitors such as azoles and macrolides may result in intolerably increased remdesivir exposure with all its ADRs. Therefore, the combination has to be avoided. Administration of potent P-gp and CYP3A4 inducers such as rifampicin and cotrimoxazole potentially decrease plasma concentrations of remdesivir and, thus, resulting in a reduction or loss of efficacy; therefore, it cannot be administered in combination. The guidance framework is still imprecise here and it is incomprehensible that a substance approved to be used in COVID-19 therapy worldwide is so understudied for interactions, as polypharmacy is often unavoidable in these patients especially.

It is of great importance to consider the same DDIs of most azoles and most macrolides, or rifampicin and cotrimoxazole, respectively, in analogous terms for nirmatrelvir/ritonavir [29]. Nirmatrelvir/ritonavir should not be started immediately after discontinuation of the antibiotics fusidic acid, rifampicin, and cotrimoxazole. This remains critical because it should be administered as early as possible with the onset of COVID-19 symptoms. Precaution is required with regard to inducer and inhibitor effects with other drugs, as these DDIs may lead to clinically significant ADRs, potentially resulting in severe, life-threatening events from greater exposures of nirmatrelvir/ritonavir due to the concomitant-inhibiting medication and vice versa; with inducers comes a loss of the therapeutic effect of nirmatrelvir/ritonavir and the possible development of viral resistance. Nirmatrelvir/ritonavir is a strong inhibitor of p-GP and CYP3A4. To give an impression of these risks, investigations with rifabutin reveal a 4-fold–2.5-fold increase, whereas the metabolite 25-O-desacetyl rifabutin has a 38-fold–16-fold increase. This large increase in the rifabutin area under the curve (AUC) requires the consequent reduction of the rifabutin dose to 150 mg 3 times per week when co-administered with ritonavir as a strong inhibitor. The ritonavir-induced, elevated AUC change of ketoconazole was 3.4-fold [29].

### 2.8. Posaconazole

Posaconazole is an azole commonly used in stem cell transplantation. Therefore, it is important to note that its inherent risk of major gastrointestinal side effects [30] with severe diarrhea may mimic or overlap CMV colitis or graft-versus-host disease, or symptomatically mask the success of their therapies and, therefore, must always be considered in differential diagnosis, especially in this accordingly most critical patient population.

### 2.9. Rifampicin

As a CYP3A4 and P-gp inducer, rifampicin, conversely, can significantly reduce the availability of CYP3A4 and P-gp substrates and, thus, their efficacy, such as in calcineurin inhibitors and mTORIs; therefore TDM and dose adjustments are required [31]. For these inducing effects, the combination with DOACs is contraindicated. There is also a wide range of DDIs resulting from inducing effects of rifampicin, e.g., with virustatics, antiepileptic drugs, and opioids, that reduce or lose their effects.

Similarly, for *metamizole*, a very common postoperative analgesic in Germany, a potential 2.9-fold hepatocyte CYP3A4 induction has been described [32], whereby a potential reduction, e.g., in the efficacy of various macrolides or azoles, cannot be excluded. We also regularly have to counteract it through dosage elevation in co-administered calcineurin inhibitors and mTORIs. Rifampicin is contraindicated in the presence of jaundice and hepatic dysfunction. Its enzyme induction can enhance the metabolism of endogenous substrates including adrenal hormones, thyroid hormones, and vitamin D. Additionally, the reported incidence of a paradoxical drug reaction ranges between 9.2 and 25%. In these cases, after the initial improvement of the tuberculosis during therapy with rifampicin, the symptoms may worsen again. An excessive immune reaction is suspected as a possible cause, which can be treated symptomatically while continuing antituberculostatic therapy [33].

### 2.10. Tigecycline

Tigecycline is known to possibly induce hyperbilirubinemia and prolonged PTT with the elevated risk of bleeding [34]. Especially in the case of concomitant continuous intravenous heparin administration, the latter often has to be adjusted according to the PTT values that need to be monitored. Based on an in vitro study, tigecycline is a P-gp substrate [34]. Co-administration of P-gp inhibitors (e.g., ketoconazole or cyclosporine) affect the pharmacokinetics of tigecycline and increase the bioavailability, including its ADRs. Concomitant P-gp inducers (e.g., rifampicin) may reduce the antibiotic effect [31]. The drug may increase serum concentrations of calcineurin inhibitors, necessitating close TDM. Tigecycline requires close monitoring for the development of superinfection, hyperbilirubinemia, hepatic injury, and pancreatitis [34].

### 2.11. Vancomycin

Thrombocytopenia is not infrequently seen with vancomycin. It is worth mentioning a reference to obese patients, whom we increasingly have to consider. Since an adjustment of the usual daily doses of vancomycin may be necessary in obese patients [35], TDM is appropriately helpful in this situation with regular level measurements because of its nephrotoxicity and further risks. Rapid bolus administration (i.e., over several minutes) may be associated with severe hypotension (including shock and rare cardiac arrest), histamine-like responses and maculopapular or erythematous rash (“red man’s syndrome” or “red neck syndrome”), and pain and spasm of the chest and spine muscles [35].

### 2.12. Voriconazole

The preclinical repeated-dose toxicity studies of the intravenous vehicle sodium beta-cyclodextrin sulfobutyl ether (SBECD) revealed vacuolization of the urinary tract epithelium and activation of macrophages in the liver and lungs as the main effects [36]. Carcinogenicity studies have not been performed with SBECD, although an SBECD-containing impurity has been shown to be a mutagenic substance with demonstrated carcinogenic potential in rodents; thus, this compound must also be considered to have carcinogenic potential in humans [36].

### 2.13. Probenecid

When used concomitantly with probenecid due to its slower excretion, the plasma levels, effect, and ADRs of several antimicrobials may be increased, and dose adjustments may be necessary in penicillin, cephalosporin, quinolone (e.g., ciprofloxacin, norfloxacin), dapsone, sulfonamides, nitrofurantoin, nalidixic acid, and rifampicin [34]. Probenecid is contraindicated in simultaneous treatment with β-lactam-antibiotics and with pre-existing renal impairment [37].

### 2.14. Proton Pump Inhibitors

Proton pump inhibitors are known to be associated with an elevated risk of bacterial infections [38]. They should be applied more restrictively, and for stress ulcer prevention, be reduced to the adequate prophylactic dosage of, e.g., daily 20 mg of pantoprazole only.

### 2.15. Benefits and Preliminary Effects of the Applied IPM Strategy

We have elaborated the domain aspects extracted and tabulated for antimicrobial ADRs and DDIs and are continuously focusing on their application in the IPM.

The unavailability despite physicians’ requests for such compilations attests to the clinical weight of this overview of risks. Studying the innumerable ADRs and DDIs from SmPCs and DDI checks takes time, which is not available in everyday clinical practice. Additionally, since countless ADRs and DDIs have emerged, especially within the broad medication list for the critically ill patients in the ICU and for polypharmacy in elderly traumatology patients who require additional antimicrobial therapy, which are impossible to comprehensively research in everyday life, we provide our insights resulting from a synoptic point of view of internal medicine and clinical pharmacology based on the daily performance of 35 to 52 IPM in an everyday real life condition by this tabulated overview. Applying this focus as one elementary building block among others of the comprehensive IPM enables us to perform a fast medication analysis in 6.5 min, whereas the extensive procedure otherwise is almost unmanageable due to the particularly broad medication lists for each patient within these settings and may take 1 to 2 h.

In addition to this benefit, the strategy employed, including maintained awareness of these knowledgeable, selective ADRs and DDIs and applying this information to each individual patient, also may contribute to the apparently highly effective IPM measures. There are compelling preliminary data suggesting that this preventive IPM strategy outlined here, documenting our IPM attention and strategic aspects, including the broad component of antimicrobials, contributes to risk reduction and optimized medication therapy in patients most vulnerable, such as those with unavoidable polypharmacy who suffer from concomitant organ dysfunction already.

Based on the annually documented reports of the controlling department of the UKH, the first preliminary descriptive results, which have to be statistically analyzed further on for corresponding publications, document that coinciding with the onset of IPM implementation for the operative ICU patients most critically ill, and for whom the ICU was the treatment ward in which thy had the longest length of stay at the UKH, the average length of stay decreased from 29.1 to 23.2 days from 2015 to 2018. In parallel, the number of patients treated even increased, e.g., by 32.7% in the ICU ward, where critically ill patients are often mechanically ventilated and dialyzed. Despite higher numbers of patients treated, medication costs concurrently decreased by 27.9% for the operative ICU wards. These results will be published separately for the entire evaluation of the IPM implementation after further analytical statistics are performed.

Regarding the department of geriatric traumatology, we retrospectively analyzed the association of the overall implemented IPM elements, including the exclusively presented, specific antimicrobial strategies with an ADR and DDI focus on prevention of complicating delirium, in-hospital fall events, and impaired renal function within a subset of 404 patients in a group-matched study. There was an IPM-associated 90.2% relative reduction in complicating delirium [16] and almost analogous results for the reduction in in-hospital fall events and in renal function impairment (for separate publication).

## 3. Discussion

IPM in the context of antimicrobial therapy reveals several clinical important aspects to consider. Most of them should be familiar as more or less typical ADRs and, thus, have not been presented here repeatedly. The selected insights into lesser-known ADRs and essential DDIs result from clinical IPM practice and observations in our patient treatments, and here, represent only excerpts from the respective ADRs and DDIs. A further number of unidentified ADRs must be assumed, since they do not always manifest in their fulminant form or a complete spectrum, but also in a crytogenic or undulating manner. This incompleteness may also apply, in part, to the identified and listed ADRs referred to in the drug product information.

Therefore, there is almost no way to definitively determine a uniform general rate of ADRs for a given drug, but the more vulnerable the patient is due to his pre-existing conditions and illnesses, organ dysfunction, and polypharmacy, the higher the risk of ADR and DDI manifestation. For this reason, post-marketing evaluations, documentations, and studies are very important because the pre-marketing trial phase of the approval studies almost never include these particularly susceptible patients.

Drugs and, especially, antibiotics can rapidly worsen an already developing single- or multi-organ failure, e.g., as a result of pre-existing hypoxia, and require special therapeutic attention with fine-tuning of dose adjustments. The essential knowledge and consideration of drug-specific ADRs, particularly in frequent antimicrobial combination therapy, is a preventive tool in patient and drug safety, both of which are continuously serious concerns as repeatedly claimed by the WHO for over a half century already [1]. A decline in renal function, and hepatic and further organ deterioration up to multi-organ failure always must exclude pharmacological iatrogenic drug-associated causation. This presentation is a sub-aspect of extensive studies of our IPM demonstrating IPM-associated optimization of medication, e.g., effectively preventing delirium by 90.2% in elderly trauma patients [16].

As ADRs are categorized inconsistently and DDIs are subsumed, this makes the important differentiation of these two risky issues less clear, and thus, the preventative, necessary countermeasures might be less appropriate or even missed.

In addition, we must take into account the most frequently prevalent hypoalbuminemia, especially in our demographically increasing elderly patient population, as well as in critically ill intensive care patients. This aspect remains almost neglected and should be focused on more intensely because, besides the predominant, clinically significant pharmacokinetic interactions observed at the metabolic level, another large proportion of competing interactions and ADR manifestations must be considered at the serum protein-binding level [39]. This further concern has not been addressed within the presented aspects. Nevertheless, we must pay additional attention to it, especially in view of the extremely common manifestation of hypoalbuminemia in our elderly and ICU patients. This can lead to severe and more rapid overexposures of high protein-binding drugs, including their ADRs. The dilemma is further exacerbated by simultaneously competing efforts of different drugs to bind to the remaining serum protein residual capacity.

It is worthwhile to depict that DDIs are of such high clinical relevance, both on the metabolic and protein-binding level, that they are even targeted in drug design and development by pharmaceutical companies. This is found with the combined therapeutic use of nirmatrelvir/ritonavir [29], and also with imipenem plus cilastin [40] and with taxanes; here, the albumin-bound nanoparticle formulation of paclitaxel and nab-paclitaxel exhibits enhanced paclitaxel tissue distribution and enhanced tumor penetration through additional active, selective transport into tumor tissue via target proteins [41,42].

Further IPM efforts are intended for communicating interventions that improve the physicians‘ recognition of these DDIs and ADRs of antimicrobials. With this in mind, we have outlined the detailed process of effective IPM enabling the early detection of ADRs and DDIs and provide the critical basis for causal, preventive medical countermeasures aimed at successful and safe antimicrobial therapy, even in the most vulnerable risk patients.

Knowledge of documented ADRs and DDIs enable early differentiation from further disease progression, which, if unrecognized, would typically and inevitably lead to additive and accumulative drug therapy to treat these unconscious iatrogenic symptoms, and thus, might be the beginning of a fatal course for the patient. The implementation of the comprehensive IPM has been found to be highly preventive of several complications, as was just published for delirium [16], and thus, IPM guarantees an important step forward in eliminating severe drug and patient safety concerns. In this context, antimicrobials are also known to potentially directly induce iatrogenic delirium, e.g., from anticholinergic properties in ampicillin, clindamycin, and gentamycin or from the dopaminergic and serotonergic agent linezolid and hyponatremia-inducing antibiotics such as cotrimoxazole [43].

In 1975, in their critical review on ADRs, Karch and Lasagna already stated, “The data on adverse drug reactions (ADRs) are incomplete, unrepresentative, uncontrolled, and lacking in operational criteria for identifying ADRs. No quantitative conclusions can be drawn from the reported data as for morbidity, mortality, or the underlying causes of ADRs, and attempts to extrapolate the available data to the general population would be invalid and perhaps misleading“ [44]. There have been no sufficiently successful attempts to overcome this serious grievance for nearly 50 years now, although it is becoming an increasingly threatening problem worldwide [45] with an inherent, extreme socioeconomic healthcare burden. Since 1998, the U.S. Food and Drug Administration has been operating the Adverse Event Reporting System, collecting all voluntary reports of adverse drug events submitted directly to the agency or through drug manufacturers. These data show a significant increase in reported deaths and serious injuries related to drug therapy during the observation period from 1998 to 2005. Within a 7-year period, the reported number of serious adverse drug events increased 2.6-fold, from 34,966 to 89,842, and the number of fatal adverse drug events increased 2.7-fold, from 5519 to 15,107 [46]. Davies et al. documented patients with adverse drug events in the hospital that had a longer length of stay and were more likely to be older, female, and taking a higher number of medications. The latter was the only significant predictor of an ADR in the multivariable analysis, with each additional medication increasing the risk of an episode of ADR by 1.14 (95% CI 1.09, 1.20) [47]. A causality and preventability assessment of adverse drug events of antibiotics among inpatients by Saqib et al. revealed 59.3% were preventable and caused by medication errors due to the nonadherence of policies (38.4%) and lack of information about antibiotics (32%) [12].

The inclusion of ADRs and DDIs as a subset of the International Classification of Diseases (ICD) is an important step to focus on these often serious to life-threatening concerns and weighting them on the level of diseases. This should be addressed more progressively. With 28.4%, Joshua et al. noted a high incidence of serious, even lethal, multiple ADRs in patients admitted to the medical ICU. Similar to our observations, they reported a broad clinical spectrum of ADRs and found infrequently documented ADRs from newer drugs. Antimicrobials (27%) were the commonly involved drugs in these ADR-susceptible patients with pre-existing multi-organ dysfunction and altered pharmacokinetic parameters [13]. In line with our IPM observations, ADRs were significantly associated with comorbidity, polypharmacy, and length of stay [13]. Severe coagulation problems and hemorrhagia, as they may occur from consumptive coagulopathy and sepsis, may also result from the ADRs and DDIs partly listed in our table. The very comprehensive IPM is a helpful tool to differentiate them more easily, particularly through its daily performance and through close patient follow-ups at the most acute, individual level.

The frequent QT prolongation risk of numerous drugs, which is often aggravated by concomitant medication in a cumulative manner, has to be regarded and is not always avoidable. Because most of the risks should be familiar, we left them out of consideration in this presentation, although the clinical consequences may become most critical. As a preventive measure, serum potassium and magnesium should be kept in an upper normal range and monitored closely, and an acidotic metabolic state should be avoided. Macrolides and azoles, most of which are highly potent inhibitors and pharmacokinetic enhancers, are antimicrobials that are important to a keep in mind, as is fluorchinolone in this context. Due to their transporter- and metabolism-inhibiting capacities, azoles and macrolides even cumulate the tachyarrhythmia risks to a higher degree than merely additively with co-administered QT prolonging drugs, which are respective substrates to the inhibited enzymes. The classification of macrolides into three different groups according to their affinity for CYP3A4, and thus, their propensity to cause pharmacokinetic drug interactions, may help to clinically estimate the severity of potentially resulting DDIs. Group 1 with troleandomycin, erythromycin, and its prodrugs decrease drug metabolism and may engender drug interactions, whereas group 2 with clarithromycin, flurithromycin, midecamycin, midecamycin acetate, josamycin, and roxithromycin should cause less frequent intense interactions [48], although according to our TDM routine data, clarithromycin is associated with a significant elevation in cyclosporine when co-administered, necessitating TDM and the timely dose adjustment of cyclosporine [49]. Additionally, the study of Hill et al. indicates that in elderly patients taking a DOAC, concomitant clarithromycin was associated with a small but statistically significant greater 30-day risk of hospital admission with major hemorrhage compared to azithromycin, a group 3 macrolide [50].

Since CYP450 enzyme generation is downregulated by elevated cytokine levels, such as from IL-1 in chronic inflammation, this would be expected to be renormalized by an IL-1 receptor antagonist such as anakinra. The clinical relevance for CYP450 substrates with a narrow therapeutic range that may be affected currently remains unknown [51]. A further aspect of interindividual varying metabolization and drug effects from mitochondrial genetics addressing personalized medicine and drug toxicity has been reviewed by Penman et al. To date, the implications remain unclear, although clinical studies have reported associations between mitochondrial haplogroup and antiretroviral therapy, chemotherapy, and antibiotic-induced toxicity. Mitochondrial DNA differences may reveal differential functions as a factor in idiosyncrasies, leading to unpredictable adverse effects and drug-induced toxicities [52]. The effect of polymorphism of the ABCB1 gene encoding P-gp was assessed by a French group for the substrates rivaroxaban and dabigatran by studying the contribution of ABCB1 genetic polymorphisms, as well as the interaction with clarithromycin, to interindividual variability under dabigatran and rivaroxaban exposure [53]. Whereas the ABCB1 genotype turned out not to be a clinically relevant determinant of both drugs’ pharmacokinetics, the co-administration of the P-gp inhibitor clarithromycin with dabigatran or rivaroxaban resulted in a clinically relevant two-fold increase in both drugs’ AUC, irrespective of the ABCB1 genotype clearly indicating the risk of DDIs [53]. With regard to almost extremely frequently administered drugs such as atorvastatin, we even find gaps in the knowledge and unawareness of significant DDIs resulting from antimicrobials. E.g., even clarithromycin (a macrolide group 2) induces a 4,5-fold increase in atorvastatin’s AUC, and for itraconazole, a 3.3-fold elevated AUC with standard dosages [54]. Given that the dual-interaction mechanism of rifampicin as an inducer of CYP3A4 and an inhibitor of the hepatocellular uptake transporter OATP1B1 is difficult to sum up already, there is even a further varying effect depending on the time of application, with a 5-fold decrease in atorvastatin when it is co-administered with rifampicin simultaneously [54].

ADRs and DDIs, as documented in antimicrobial therapy from our IPM insights, are an increasing challenge in today’s healthcare worldwide, especially given the growing complexity of chronic and acute combination therapy interventions, interdisciplinary treatments by different specialists, an aging population with increasing multimorbidity, and additionally, disease interactions from renal impairment or hepatic dysfunction. An alarming deficit of ADR and DDI awareness is burdening the patients, the hospitals, the entire healthcare sector, and the public budget. Urgent intervention strategies for prevention are to be promoted and anchored politically. This may be achieved via the development of increasingly more effective digital medication tools and making institutions of specialized drug safety managers mandatory in hospitals and health insurance companies to cover the inpatient and outpatient care sectors.

## 4. Methods

This study from ongoing, real-life observations was designed to summarize and tabulate the under-recognized, clinically most critical risks of ADRs and DDIs in the context of current medication regimens and in the setting of elderly, multimorbid, or even seriously ill hospitalized patients. Critical, but less recognized, ADR and DDI risks from daily 35–52 medication reviews were selected through comprehensive individual pharmacotherapy management (IPM) in these vulnerable operative intensive care, elderly traumatology, and organ and stem cell transplant patients. The underlying observation period was from 12/2014 to 8/2022. We examined the drug combinations found in current, real-life practice based on medication reviews of >52,000 IPMs. As a consequent part of the study, we also provide examples of our own practice-proven advice on control and countermeasures.

IPM was designed and implemented by a University Hospital Halle (UKH) physician with expertise in internal medicine and clinical pharmacology, and who is responsible for the pharmacotherapy management at the UKH in continuous cooperation with senior physicians of the operative intensive care units (ICUs) and geriatric traumatology. The focus is on patient and drug safety, especially in these most vulnerable groups with polypharmacy, often additionally associated with impaired organ function, as in interdisciplinary intensive care patients, organ or stem cell transplanted patients, and elderly trauma patients. IPM considers the entire digital patient record from the internal clinical information systems for data management, the Integrated Care Manager (ICM) and Orbis software for advice on clinical documentation. Always in synopsis internal medicine/clinical pharmacology, the comprehensive medication review has been performed by the same study physician for each patient daily in the operative ICUs and fortnightly for interdisciplinary patient visitations in geriatric traumatology. The review was conducted primarily based on the Summary of Product Characteristics (SmPC), particularly considering pharmacokinetics, pharmacodynamics, dosage, contraindications, interactions, and all ADRs. In addition, the literature research was carried out to answer extended or unresolved questions. All diagnoses and indications were recorded as the basis for each individual medication review. Further on, current medical guidelines were taken into account.

Recommendations result from the entire consideration of the individual patient’s situation, both regarding his chronic and current clinically relevant disease process, and the acute pre- and postoperative situation, considering all available laboratory medical data on organ functions; albumin; lactate and inflammation parameters; electrocardiography and imaging examination results; the continuous course of vital parameters (blood pressure, heart rate, respiratory function, and acid–base balance); body weight; BMI; cognitive disorders; other subjective complaints; pain intensity/profile; the results of assessments and risk scores; demographic data such as age, gender, and living situation; anamnesis/external anamnesis; and the course of clinical examination findings. There is always an additional focus on anticholinergic components and electrolyte disturbances, glucose metabolism, vitamin D, parathormone balance and endocrinological thyroid functions, coagulation parameters, transient organ replacement therapies, and manifest/potential hematologic disorders, in particular, on all forms of anemia. This comprehensive digital overall view of a patient represents the basis for the internistic/clinical pharmacologic intervention with the simultaneous synoptic and adaptive examination of all medications.

By the consequent implementation of this regularly systematic individual pharmacotherapy management, the focus is on prevention and elimination of iatrogenic medication-associated: 1. severe hypotension from cumulative drug effects, 2. renal injury, 3. single and multiple organ failure, 4. neurologic/psychiatric disorders including delirium, 5. in-hospital fall events, 6. arrhythmias, 7. hemorrhages from hemostaseologic disorders, 8. cerebrocardiovascular events, 9. hematologic/myeloproliferative disorders, 10. all types of differentiated anemias, 11. venous thrombosis, 12. acid–base and electrolyte imbalance, 13. metabolic/endocrinologic disorders, 14. gastrointestinal effects such as paralytic ileus and ulcer of the upper intestine, 15. multidrug-resistant nosocomial infections, and 16. oropharyngeal dysphagia.

In this context, during the 8 year follow-up of IPM we recognized risks of ADRs and DDIs resulting from antimicrobials, which are of predominant clinical relevance concerning the regarded aspects. As we obviously work with a similar time pressure as most physicians nowadays do, we, exactly for this reason, sum up and compile into a table for overview the most critical risks resulting from our clinical insights of the comprehensive IPM, that always adapts drug information to the individual and acute patient clinical condition.

The findings presented here are a specific sub-focus of lesser referenced ADRs and relevant DDIs associated with the concomitant administration of antimicrobials in the context of our IPM. We explicitly exclude the more familiar nephrotoxic risks, QT prolongation, and myelosuppression as the most typical ADRs of several antimicrobial agents. We covered the relevant pharmacological aspects with which we are not so familiar to provide additionally corresponding further practical guidance through IPM.

## 5. Strengths and Weaknesses

The analysis refers to continuous observations on selected wards of the UKH. Nevertheless, the aspects addressed may be representative for the entire patient population. IPM is an individual medication review that is conducted as intensively as possible. Genetic pharmacological aspects are not considered additionally. We did not measure blood values that attribute adverse effects to presumably iatrogenic medical agents due to elevated levels. Only from the time of occurrence during application and reversal of the critical situation by selective drug withdrawal could a possible causal relationship be postulated. However, the causal association remains difficult to definitively prove due to numerous pathophysiological disturbances often occurring in parallel in our severely ill patients. The more than 8 years of daily IPM experience with a critically ill or multimorbid elderly interdisciplinary patient population covers a particularly vulnerable group with polypharmacy and, probably, already reduced organ function. There is almost no possibility to clearly identify the entire frequency of ADR manifestation, but the more vulnerable the patient is as a result of pre-existing illnesses, organ deterioration, and polypharmacy, the higher the ADR risk. The spectrum of ADRs and DDIs addressed is based primarily on the SmPCs, whose completeness and, in rare cases, even the presentation of clearly defined metabolic pathways of active substances and risks from inactive metabolites are not always fulfilled, thus leaving out still unknown grey areas, which may be additionally dangerous for the treated patients.

## 6. Conclusions and Outlook

IPM-based awareness of serious but lesser-known ADRs and DDIs of antimicrobial agents can counteract or reverse single- or multi-organ failure, cardiac arrhythmias, life-threatening hemorrhage or thrombosis, hematologic/myeloproliferative disorders, electrolyte imbalances, neurologic/psychiatric disorders, and drug escalations by enabling timely and targeted correction of the underlying causation individually. The conceptionalized IPM strategy applied to optimize drug treatment and prevent ADRs and DDIs, both common risks of polypharmacy, is accompanied by a reduction in length of hospital stay and associated costs. We plan to implement IPM’s longstanding experience and lessons learned for wider use on a digitized platform to share a comprehensive compass, in addition to the documented findings on antimicrobials, as an important contribution to improving patient and drug safety collectively when facing the high-risk issue of under-recognized ADRs and DDIs worldwide.

## Figures and Tables

**Table 1:** Under-recognized potential ADRs and DDIs of clinical relevance from IPM insights with primary reference to their Summaries of Product Characteristics. This table is intentionally incomplete and excludes the more familiar gastrointestinal, nephrotoxic, several QT prolonging, several cutaneous dermatologic, and all anaphylactic and further risks.

ADR and DDI Antimicrobial	Neurologic/ Psychiatric	Hematologic	Hemostaseologic	Endocrinologic/ Metabolic	Cardiac	Others
**Anidulafungin**	Convulsions			Hypokalemia Hyperglycemia		Hepatic dysfunction
**Azoles**			Contraindicated with all currently available DOACs and ticagrelor, severe risk of hemorrhage		QT prolongation and tachyarrhythmia Reduces amiodarone metabolism and vice versa	Risk of rhabdomyolysis with atorvastatin, lovastatin, simvastatin Increase in blood levels and effects of calcineurin inhibitors and mTORIs Increase in bioavailability and effects of several virustatics, e.g., remdesivir * and nirmatrelvir/ritonavir for COVID-19 therapy
**Carbapenems**	No concomitant use of valproic acid/sodium valproate/valpromide: 60–100% decrease in valproic acid levels in about two days Hallucinations, dizziness, seizures Generalized seizures Imipenem/cilastin and ganciclovir	Thrombocytosis Thrombocytopenia		High-sodium content		Hepatic dysfunction Probenecid (potent inhibitor of organic anion secretion by renal proximal tubule cells) inhibits renal excretion
**Cotrimoxazole**		Leukopenia, neutropenia, thrombocytopenia, agranulocytosis, anemia: megaloblastic, aplastic anemia hemolytic, possible folic acid deficiency anemia Folic acid deficiency		Hyperkalemia Hyponatremia Metabolic acidosis Note thyroid function		Hepatobiliary disorders Life-threatening cutaneous reactions Enhanced effect with probenecid, sulfinpyrazone, indometacin, phenylbutazone, salicylate Reduced serum cyclosporine concentrations with risk of transplant rejection
**Daptomycin**	Anxiety, insomnia, dizziness, headache	Anemia	False prolongation of prothrombin time (PT) and elevation of international normalized ratio (INR) with certain recombinant thromboplastin reagents			Other medicinal productsassociated with myopathy should be temporarily discontinued, creatine phosphokinase (CPK) levels must be measured at baseline and at regular intervals Elevated transaminase levels NSAIDs and COX-2 inhibitors elevate plasma levels Eosinophilic pneumonia Obesity increases daptomycin mean AUC0-∞ In elderly patients, AUC0-∞ about 58% higher
**Fluoroquinolones**	Psychomotor hyperactivity/ agitation, confusion, depression			Hyperglycemia	Inherent risk of QT prolongation is increased with a broad spectrum of drugs such as macrolides, azoles, antipsychotics, class IA and III antiarrhythmics, tricyclic antidepressants Tachycardia Risk of valvular regurgitation/insufficiency	Ciprofloxacin may increase cyclosporine (CSA) bioavailability and elevate the risk of CSA-induced neurotoxicity and nephrotoxicity Aneurysm, aortic dissection, tendon rupture Myalgia Inhibits via CYP1A2 the metabolism of theophylline, clozapine, olanzapine, ropinirole, tizanidine, duloxetine Contraindicated with tizanidine Contraindicated with methotrexate Hepatic dysfunction
**Linezolid**	Cave: linezolid is a reversible, nonselective inhibitor of monoamine oxidase (MAOI) Serotonin syndrome associated with the coadministration of serotonergic agents Neuropathy (optic and peripheral) Neither with inhibitors of monoamine oxidase A (e.g., phenelzine) or B inhibitors (e.g., phenelzine, isocarboxazid, selegiline, moclobemide), nor with one of these drugs taken within the last 2 weeks Malignant neuroleptic syndrome Taste disorders (metallic taste), dizziness, insomnia	Thrombocytopenia, anemia, pancytopenia		Lactate acidosis Hyponatremia and/or syndrome of inappropriate secretion of antidiuretic hormone (SIADH) Hyperglycemia (not fasting blood sugar)		Not to be used, unless monitoring of blood pressure in patients with uncontrolled hypertension, pheochromocytoma, carcinoid, thyrotoxicosis, bipolar depression, schizoaffective psychosis, acute confusion conditions Not with serotonin reuptake inhibitors, tricyclic antidepressants, serotonin 5HT1 receptor agonists (triptans), direct- or indirect-acting sympathomimetics (including adrenergic bronchodilators, pseudoephedrine and phenylpropanolamine), vasopressor agents (e.g., epinephrine, norepinephrine), dopaminergic agents (e.g., dopamine, dobutamine), pethidine, or buspirone, high-tyramine foods Increased transaminases, lipase, CPK, LDH
**Macrolides** **Cave: three categories to be differentiated in terms of their interaction characteristics**			Contraindicated with all currently available DOACs, ticagrelor, severe risk of hemorrhage		QT prolongation and tachyarrhythmia Reduced amiodarone metabolism and vice versa Reduced azole metabolism and vice versa	Risk of rhabdomyolysis with atorvastatin, lovastatin, simvastatin Increase in blood levels and effects of calcineurin inhibitors and mTORIs Increased bioavailability and effects of several virustatics, e.g., remdesivir * and nirmatrelvir/ritonavir for COVID-19 therapy
**Piperacillin/** **Tazobactam**	Insomnia		Activated partial thromboplastin time and bleeding time prolonged	Hypernatremia: 217 mg of sodium per vial of powder for solution for infusion		In elderly patients, mean half-life for piperacillin and tazobactam was 32% and 55% longer
**Rifampicin**			Reduces plasma level and effects of all currently available DOACs and ticagrelor Vitamin K-dependent coagulation and severe bleeding Supplemental vitamin K administration should be considered when appropriate (vitamin K deficiency, hypoprothrombinemia) Avoid concomitant use of rifampicin with other antibiotics causing vitamin K-dependent coagulopathy such as cefazolin (or other cephalosporins with N-methyl-thiotetrazole side chain) as it may lead to severe coagulation disorder	Reduces bioavailability of oral contraceptives Enzyme induction with enhanced metabolism of endogenous substrates as adrenal hormones, thyroid hormones, and vitamin D	Rifampicin strongly induces CYP2C19, resulting in both an increased level of clopidogrel active metabolite and platelet inhibition, which, in particular, might potentiate the risk of bleeding; avoid combination	Caution in elderly patients with decrease in renal function, especially if there is evidence of liver function impairment Severe cutaneous adverse reactions (SCARs) Reduced blood levels and effects of calcineurin inhibitors and mTORIs with risk of transplant rejection Reduced bioavailability and effects of several virustatics, e.g., remdesivir * and nirmatrelvir/ritonavir for COVID-19 therapy Loss of efficacy of caspofungin, dosage increase in caspofungin necessary Contraindicated with voriconazole (treatment failure), reduced effects of azoles Reduced or loss of effect of a broad spectrum of drugs including opioids, calcium antagonists, etc.
**Tigecycline**			Prolongation of prothrombin time (PT) and activated partial thromboplastin time(aPTT), hypofibrinogenemia			Close monitoring for the development of superinfection Hyperbilirubinemia, hepatic injury, pancreatitis P-gp substrate Cave: inhibitors such as azoles or macrolides enhance bioavailability and effects and P-gp inducers such as rifampicin decrease tigecycline bioavailability and effects Increases serum concentrations of calcineurin inhibitors
**Vancomycin**	Ototoxicity, periodic testing of auditory function	Thrombocytopenia, neutropenia, agranulocytosis, eosinophilia			Anesthetic-induced myocardial depression may be enhanced by vancomycin	Monitoring of serum concentrations Adapted according to weight, age, and renal function “red man’s syndrome” Dyspnea, stridor

* Note: Despite partly confusing published guidance for remdesivir on the management of its potential drug interactions, it is critical to know that the interaction potential and clinical relevance have not been studied yet, although in vitro remdesivir is a substrate for esterases in plasma and tissue, for the metabolic enzymes CYP2C8, CYP2D6, and CYP3A4, and for the organic anion-transporting polypeptide 1B1 (OATP1B1) and the P-glycoprotein transporter. In vitro, remdesivir is an inhibitor of CYP3A4, OATP1B1, and OATP1B3 and remdesivir induces in vitro CYP1A2 and, potentially, CYP3A, which is partially contradictory.

## Data Availability

There was no data collection for the purpose of this article, only observational results and recommendations.
